# Draft Genome Sequence of *Legionella jamestowniensis* Isolated from a Patient with Chronic Respiratory Disease

**DOI:** 10.1128/genomeA.01007-16

**Published:** 2016-09-15

**Authors:** Birgit Prochazka, Alexander Indra, Petra Hasenberger, Marion Blaschitz, Laura Wagner, Günther Wewalka, Sieglinde Sorschag, Daniela Schmid, Werner Ruppitsch

**Affiliations:** a Institute of Medical Microbiology and Hygiene, Austrian Agency for Health and Food Safety, Vienna, Austria; b Department of Hospital Hygiene and Infectious Diseases, Community-Hospital Klagenfurt am Wörthersee, Klagenfurt, Austria; c Department of Biotechnology, University of Natural Resources and Life Sciences, Vienna, Austria

## Abstract

*Legionella jamestowniensis* can be found in the environment in various water samples, in wet soil, and in compost facilities, but evidence of its human pathogenicity has not yet been demonstrated. Here, we report the first draft genome sequence of an *L. jamestowniensis* isolate, derived from a patient suffering from a chronic respiratory disease.

## GENOME ANNOUNCEMENT

Legionellae are Gram-negative bacteria inhabiting aquatic environments or wet soil and survive as intracellular parasites of amoebae and ciliates (1–3). The genus *Legionella* includes 39 species (4), of which 19 have been implicated in human disease (5). *L. jamestowniensis* was isolated for the first time in 1985 in Jamestown, New York, USA, from wet soil (6). Since then, *L. jamestowniensis* has been isolated from various water samples, wet soil, and compost facilities (7). *L. jamestowniensis* has not been associated with human disease so far, but it can infect human cells (5). An *L. jamestowniensis* isolate was obtained from a patient with clinical signs of acute bronchitis in 2012 in Austria. The patient had a long-term history of about 20 years of recurrent bronchitis, asthma, and bronchiectasis. The findings of bronchoscopy, histopathological, cytological, and serological examinations indicated an allergic bronchopulmonary aspergillosis. No indications for tuberculosis and no culturable mycobacteria could be found. The microbiological laboratory of the hospital could cultivate a *Legionella* isolate of an unidentifiable species. The Austrian National Reference Laboratory for Legionella Infections identified the isolate as *L. jamestowniensis*, with MALDI-TOF score values of 1,997 and 1,971 (100% mip sequence concordance to *L. jamestowniensis* ATCC 35298), and a tetra correlation search (TCS) score of 0.9998 to the *L. jamestowniensis* database reference sequence using JSpeciesWS (8). This is the first reported draft genome of *L. jamestowniensis* derived from a patient.

The MagAttract HMW DNA kit (Qiagen, Hilden, Germany) was used to isolate genomic DNA from an overnight culture grown on *Legionella*-selective agar plates with growth and antibiotic supplements (Oxoid). The fragment library was prepared using Illumina’s NexteraXT kit (Illumina Inc., San Diego, CA, USA) and 1 ng of genomic DNA. Paired-end sequencing (2 × 300-bp) was performed on a MiSeq (Illumina Inc.), generating 1,953,138 reads from 575,452,070 unassembled nucleotides. Raw reads were *de-novo* assembled into a draft genome using Velvet version 1.1.04 (9). Contigs were filtered for a minimum coverage of 5× and minimum length of 200 bp, which resulted in 53 contigs with a total of 1,953,138 nucleotides at a coverage of 108×; 2,945 genes, 2,895 coding sequences, 58 pseudogenes, 50 rRNA genes, 42 tRNA genes, and four ncRNAs were identified by the NCBI Prokaryotic Genome Automatic Annotation Pipeline.

### Accession number(s).

This whole-genome shotgun project has been deposited at DDBJ/EMBL/GenBank under the accession number LYOZ00000000. The version described in this paper is the first version, LYOZ01000000.
